# Supplementary material for: Complexome profiling of the Chlamydomonas psb28 mutant reveals TEF5 as an early PSII assembly factor
Source: Plant Cell. 2025 Mar 17;37(6):koaf055. doi: 10.1093/plcell/koaf055 (PMC12164590; doi:10.1093/plcell/koaf055)
Supplement: koaf055_Supplementary_Data [file koaf055_supplementary_data.zip › psb28_interactive.zip!/plots/intensity/qualityPlots/peptides/_Cre09.g413750.t1.1.html]

### Cre09.g413750.t1.1

Trivial name:   
SeqCoverage [median]: 9.60  
Mass: 50.30 kDa  
  
 

| Sequence | MissedCleavage | Mass [Da] | wtIntensity | muIntensity | Score |
| --- | --- | --- | --- | --- | --- |
| GIASGYPLAGVATR | 0 | 1331.72 | 440.64 | 2840.41 | 101.95 |
| LASLLAGGTASAAR | 0 | 1257.70 | 0.00 | 2664.70 | 72.20 |
| LTDNAQER | 0 | 945.45 | 692.79 | 883.62 | 111.95 |
| TLGAMALTSSK | 0 | 1078.57 | 0.00 | 586.98 | 66.19 |
|  |  |  | 1133.4 | 6975.7 |  |
